# Gender Disparity Impacts on Thymus Aging and LHRH Receptor Antagonist-Induced Thymic Reconstitution Following Chemotherapeutic Damage

**DOI:** 10.3389/fimmu.2020.00302

**Published:** 2020-03-03

**Authors:** Michael Ly Hun, Kahlia Wong, Josephine Rahma Gunawan, Abdulaziz Alsharif, Kylie Quinn, Ann P. Chidgey

**Affiliations:** ^1^Thymus Development, Ageing and T Cell Regeneration Laboratory, Department of Anatomy and Developmental Biology, Biomedicine Discovery Institute, Monash University Clayton, Melbourne, VIC, Australia; ^2^Quinn Laboratory, Translational Immunology and Nanotechnology Research Program, School of Health and Biomedical Research, RMIT University, Melbourne, VIC, Australia

**Keywords:** thymus, thymic epithelial cell, aging, gender, sex hormone deprivation, luteinizing hormone-releasing hormone, chemotherapy, regeneration

## Abstract

One of the main consequences of thymus aging is the decrease in naïve T cell output. This condition accelerates at the onset of puberty, and presents as a major clinical complication for cancer patients who require cytoablative therapy. Specifically, the extensive use of chemotherapeutics, such as cyclophosphamide, in such treatments damage thymic structure and eliminate the existing naïve T cell repertoire. The resulting immunodeficiency can lead to increased incidence of opportunistic infections, tumor growth relapse and/or autoimmune diseases, particularly in older patients. Thus, strategies aimed at rejuvenating the aged thymus following chemotherapeutic damage are required. Previous studies have revealed that sex hormone deprivation in male mice is capable of regenerating the thymic microenvironment following chemotherapy treatment, however, further investigation is crucial to identify gender-based differences, and the molecular mechanisms involved during thymus regeneration. Through phenotypic analyzes, we identified gender-specific alterations in thymocytes and thymic epithelial cell (TEC) subsets from the onset of puberty. By middle-age, females presented with a higher number of thymocytes in comparison to males, yet a decrease in their Aire^+^ medullary TEC/thymocyte ratio was observed. This reduction could be associated with an increased risk of autoimmune disease in middle-aged women. Given the concurrent increase in female Aire^+^ cTEC/thymocyte ratio, we proposed that there may be an impediment in Aire^+^ mTEC^hi^ differentiation, and Aire^+^ cTEC^hi^ as its upstream precursor. The regenerative effects of LHRH receptor antagonist, degarelix, on TEC subsets was also less pronounced in middle-aged females compared to males, possibly due to slower progression of thymic involution in the former, which presented with greater TEC^hi^ proportions. Furthermore, following cyclophosphamide treatment, degarelix enhanced thymocyte and mature TEC subset recovery, with faster recovery kinetics observed in females. These events were found to involve both reactivation and proliferation of thymic epithelial progenitor cells. Taken together, the findings from this study portray a relationship between gender disparity and thymus aging, and highlight the potential benefits of LHRH receptor antagonist treatment for thymic regeneration. Further research is required, however, to determine how gender may impact on the mechanisms underpinning these events.

## Introduction

Systemic chemotherapy regimens are commonly used to eradicate malignant tumors, but do so at the cost of harming other normal rapidly dividing cells. These include cells found within hair follicles, mucous membranes and the hematopoietic compartment. Such treatment regimens result in patient susceptibility to opportunistic infections, a consequence of immunodeficiency from T lymphocyte depletion and delayed repopulation (1). Given the increased risk of morbidity and mortality in aged cancer patients, it is clear that prompt immune reconstitution is paramount.

Cyclophosphamide is an alkylating agent that disrupts DNA replication to prevent cell proliferation (2). This commonly used chemotherapy reagent, like others, causes the thymus to transiently involute (3). In conjunction with the adverse effects of age-related thymic atrophy, the stromal damage induced by chemotherapy acts as an impediment to T cell reconstitution (4). Specifically, it has been shown that cyclophosphamide treatment in young male mice depleted the mature autoimmune regulator (Aire)^+^ medullary (m) thymic epithelial cell (TEC) population, which is important for central tolerance (5). This event presents the possibility of autoimmune reactive T cell escape (6). Furthermore, within the first couple of years following chemotherapy, peripheral T cell recovery in adult patients has been shown to result from clonal expansion of resistant memory T cells, rather than repopulating naïve T cells, that were later found to be susceptible to apoptosis (7).

Age-related thymic atrophy influences the kinetics of naïve T cell recovery following cytoablative damage. This degenerative process impedes naïve T cell output, with the functional epithelial compartment of the thymus progressively replaced by adipose (8, 9). These effects are most pronounced from the onset of puberty, where the increased production of sex steroids and sudden drop in thymocyte number parallels a proportional loss of mature mTECs (10). Moreover, the direct effects of sex hormone receptor activation has been demonstrated, with a sudden decline in TEC number and deterioration of naïve T cell output observed following testosterone treatment (11, 12).

Despite the importance of mature mTECs for self-tolerance induction, their aged-related decline is less consequential during healthy normal life. Postnatal, single lineage mTEC progenitors can somewhat maintain the mature mTEC compartment (13), whilst Foxp3^+^ T regulatory cells compensate for the escape of potentially autoreactive T cells (14). However, following thymic damage and peripheral T cell loss, such as from multiple-dose chemotherapy and irradiation regimens, older patients with an atrophied thymus have limited capacity to regenerate naïve T cells. This results in a severely restricted range of antigen specificities in the patient's T cell repertoire, and hence increased morbidity and mortality. Thus, *de novo* reconstitution of a diverse naïve T cell pool would be optimal for robust and sustained immune recovery.

The increased quiescence of a bipotent thymic epithelial progenitor cell (TEPC) subpopulation from the onset of puberty (10) possibly underlies the greater reliance on single lineage mTEC^lo^ precursors to maintain the postnatal mature mTEC^hi^ compartment (13). It is therefore possible that thymus recovery can potentially be achieved through the reactivation of adult TEPCs (15–18). Moreover, the age-induced alterations in thymic follistatin (Fst) and bone morphogenetic protein 4 (Bmp4) production have been proposed to play a role in the reduced differentiation of thymic epithelial progenitors into their downstream mature TEC counterparts via inhibition of activin A signaling (10). As such, modulation of this and other signaling cascades may also prove beneficial to ultimately achieve T cell reconstitution.

Sex hormone deprivation (SHD) via reversible chemical castration has been considered as a possible clinical strategy for thymus regeneration in immunodeficient patients (12). The most notable agents for chemical castration are gonadotropin-releasing hormone (GnRH) or luteinizing hormone releasing hormone (LHRH) analogs. These agents are commonly used in the treatment of breast cancer and metastatic prostate cancer, with clinical studies assessing immune regeneration in prostate cancer patients demonstrating improved T cell responses (19–21). In many pre-clinical studies, SHD has been shown to transiently reverse immune aging, rejuvenating both the B and T lymphocyte arms of adaptive immunity (11, 19, 22–26). A numerical increase in bone marrow-derived early thymic progenitors (ETPs), B lymphocytes and lineage-negative Sca-1^+^ c-kit^+^ (LSK) cells was observed with SHD following chemotherapy and hematopoietic stem cell transplantation. Furthermore, SHD via LHRH receptor agonist treatment has been shown to significantly augment thymic recovery (22, 27), with LHRH receptor antagonism promoting thymopoiesis more rapidly than their agonist counterparts, and facilitating increased *Delta-like 4* (*Dll4*) expression in cortical (c) TECs (12). Since LHRH receptor antagonists circumvent the initial spike in sex steroid production caused by LHRH receptor agonists, which may cause further thymic damage, they represent a potentially superior approach when considering immune regeneration. Whether or not LHRH analog-induced regeneration of the aged thymus provides adequate self-tolerance mechanisms has been questioned (28), however, a more recent publication suggests regeneration of the mTEC compartment does occur at least in the middle-aged thymus (10). Moreover, the majority of pre-clinical research into thymic involution and sex steroid inhibition-induced thymic reactivation has been conducted in male mice. It is hence clear that further investigation is required to identify whether gender disparity exists in the loss of TEC compartments during aging and to determine whether similar mechanisms govern thymus regeneration in females.

Here, we build upon our previous research to investigate the role of postnatal bipotent TEPCs in thymus aging and damage recovery (10, 18). Specifically, we examine middle-aged female and male mice for associations between sexual dimorphism and TEC loss with aging, endogenous TEC regeneration following single dose cyclophosphamide treatment, and potential for enhanced thymic restoration following administration of the LHRH receptor antagonist, degarelix.

## Materials and Methods

### Animals

C57BL/6J mice (pre-pubertal, 4-week-old; post-pubertal, 7-week-old; and middle-aged, 7–12-month-old) were obtained from Monash Animal Research Platform and housed at Animal Research Laboratory (Monash University, Australia). Mice were maintained in a controlled environment with a standard diet and water *ad libitum*. All experiments were conducted according to Australian National Health and Medical Research Council Guidelines of Animals Used for Scientific Purposes (2008), and were approved by Monash University Animal Ethics Committee (SOBSA/ADB/2015/039).

### LHRH Receptor Antagonist and Chemotherapy Treatment

For all experiments, the final day of treatment was designated as day 0 (D0). Mice were treated with degarelix (Firmagon® LHRH receptor-antagonist) at a dose of 78 μg/g, injected subcutaneously 48 h prior (D-2) to allow time for estrogen and testosterone production to reach castrate levels by D0. Cyclophosphamide (Endoxan®) in mouse tonicity phosphate-buffered saline (PBS) was injected intraperitoneally over two consecutive days at 0.1 mg/g/day (D-1 and D0) to simulate its clinical application. Mice were subsequently analyzed following euthanasia through CO_2_ asphyxiation at indicated time points.

### Enzymatic Digestion of Thymic Tissue

Following thoracotomy, thymi were collected in RPMI medium 1640 (Gibco, U.S.A.), and each thymus cleaned of connective tissue. Thymi were then snipped with fine scissors, and enzymatically digested using 0.02% (w/v) DNase I and 0.0185% (w/v) Liberase Thermolysin Medium (Roche, Germany) in RPMI medium 1640, for 15 min at 37°C (29). Thymic fragments were gently agitated using a wide-bore pipette tip and allowed to settle before collection of the supernatant. Fluorescence-activated cell sorting (FACS) buffer (0.1% BSA and 5 mM EDTA in PBS), was added to neutralize enzymatic activity. The remaining fragments were then digested with fresh enzyme, and the cycle repeated until completion. Smaller pipette tips were used for agitation as digestion progressed. Lastly, filtration was performed on pooled thymic fractions through a 100 μm nylon mesh, followed by centrifugation at 500 g_*max*_ for 3 min at 4°C. Cell pellets were resuspended in FACS buffer, and cell counts acquired using a Z2 Coulter Counter (Beckman Coulter, U.S.A.).

### Preparation of Lymph Nodes for T Cell Analysis

Lymph nodes (bilateral brachial and inguinal) were dissected and mechanically digested using two frosted glass slides in FACS buffer to create a single cell suspension. Cells were filtered through a 100 μm nylon mesh before counting using a Z2 Coulter Counter. A Z2 Coulter Counter was then used to determine total and viable cell numbers prior to immunostaining for flow cytometric analysis.

### Flow Cytometric Analysis

Cells were resuspended in primary antibody cocktail at a concentration of 1 × 10^6^ cells per 10 μl (minimum 20 μl), and incubated in the absence of light for 15 min at 4°C. Unbound antibodies were removed by washing with FACS buffer. Following centrifugation at 500 g_*max*_ for 3 min, cell pellets were stained with secondary antibody (where appropriate) for 15 min at 4°C. Stained samples were washed and resuspended in FACS buffer after centrifugation, then filtered into round-bottom polystyrene tubes. Lastly, propidium iodide (PI; Sigma Aldrich, U.S.A.) was added into each sample tube to exclude dead cells for live stain analyzes.

Intracellular staining was performed to identify cell proliferation (Ki-67), Aire, and Foxp3 expression in TEC and/or T cell subpopulations. Cells previously stained with extracellular markers were fixed using Cytofix™ buffer (eBioscience, U.S.A.) for 30 min at 4°C, according to the manufacturer's instructions. Samples were subsequently washed with Perm-wash buffer (BD Biosciences, U.S.A.), centrifuged at 500 g_*max*_ for 3 min, and stained with intracellular antibodies or their isotype controls for 30 min at 4°C. Stained cells were washed, resuspended in FACS buffer following centrifugation, and transferred into round-bottom tubes for flow cytometric analysis (29).

Stained cell samples were acquired using a BD FACSCanto™ II flow cytometer (BD Bioscience, U.S.A.). Parameter, voltage and compensation settings were established using BD FACSDiva v.6 software (BD Bioscience, U.S.A.). Data were analyzed using FlowLogic™ v700.1A (Inivai Technologies, Australia).

Antibodies used for immunofluorescent staining are listed below (Table 1).

**Table 1:** Antibodies utilized for immunofluorescent staining—flow cytometric analysis.

**Antibody**	**Marker**	**Host/isotype/clone**	**Supplier**
CD45	Thymocyte	Rat IgG2b, κ 30-F11	BD Biosciences, U.S.A.
TER119	Erythrocyte	Rat IgG2b, κ TER-119	BD Biosciences, U.S.A.
EpCAM	Epithelial	Rat IgG2a, κ G8.8	Biolegend, U.S.A.
UEA-1	Medullary TEC	Lectin	Vector Labs, U.S.A.
Ly-51	Cortical TEC	Rat IgG2a, κ 6C3	BD Biosciences, U.S.A.
MHCII	TEC maturity	Rat IgG2b, κ M5/114.15.2	Biolegend, U.S.A.
α6-integrin	TEPC	Rat IgG2a, κ GoH3	Biolegend, U.S.A.
Sca-1	TEPC	Rat IgG2a, κ D7	eBioscience, U.S.A.
Ki-67	Proliferation	Mouse IgG1, κ B56	BD Biosciences, U.S.A.
Aire	Autoimmune regulator	Rat IgG2c, κ 5H12	Invitrogen, U.S.A.
Streptavidin	Biotin	Protein	BD Biosciences, U.S.A.
CD44	Activated T-cell	Rat IgG2b, κ IM7	eBioscience, U.S.A.
CD25		Rat OFA IgG1, λ PC61	BD Biosciences, U.S.A.
CD117		Rat IgG2b, κ 2B8	eBioscience, U.S.A.
CD3ε	Thymocyte	Ar hamster IgG1, κ 145-2C11	BD Biosciences, U.S.A.
CD4	Helper T-cell	Rat IgG2b, κ RM4-5	BD Biosciences, U.S.A.
CD8	cytotoxic T-cell	Rat LOU IgG2a, κ 53-6.7	BD Biosciences, U.S.A.
CD19		Rat LEW IgG2a, κ 1D3	BD Biosciences, U.S.A.
CD11b		Rat IgG2b, κ M1/70	BD Biosciences, U.S.A.
CD11c		Ar Hamster IgG1, λ2 HL3	BD Biosciences, U.S.A.
Gr-1/Ly-6G		Rat IgG2b, κ RB6-8C5	BD Biosciences, U.S.A.
CD45R/B220	B-cell	Rat IgG2a, κ RA3-6B2	BD Biosciences, U.S.A.
NK1.1		Mouse IgG2a, κ PK136	BD Biosciences, U.S.A.
H-2kb		Mouse BALB/c IgG2a, κ AF6-88.5	BD Biosciences, U.S.A.
CD62L	Naïve T-cell	Rat IgG2a, κ MEL-14	eBioscience, U.S.A.
Foxp3		Rat IgG2a, κ FJK-16s	eBioscience, U.S.A.
TCRβ	T-cell	Ar Hamster IgG2, λ1 H57-597	BD Biosciences, U.S.A.
CD49d		Rat IgG2b, κ R1-2	Biolegend, U.S.A.
CD122		Rat SD IgG2b, κ TM-β1	BD Biosciences, U.S.A.

### Thymic Stromal Cell Isolation

To enrich for CD45^−^ thymic stromal cells, anti-mouse CD45 MicroBeads (Miltenyi Biotec, Germany) were added to pooled thymic digests (5 μl beads + 95 μl FACS buffer per 1 × 10^7^ cells) and gently rotated for 20 min at 4°C. Samples were washed with FACS buffer and centrifuged at 300 g_*max*_ for 10 min to remove unbound magnetic beads, then resuspended in FACS buffer at a concentration of 0.5 × 10^8^ cells/ml. Isolation of CD45^−^ fractions was achieved using the “Deplete” function of an AutoMACS Pro Separator (Miltenyi Biotec, Germany). The purified thymic stromal cells were then incubated in RBC lysis buffer for 2 min at 37°C and resuspended in FACS buffer for immunofluorescent staining (as described in Thymic Stromal Cell Isolation). Stained CD45^−^ thymic stromal cell subsets were subsequently sorted with a BD Influx™ I cell sorter (BD Bioscience, U.S.A) at FlowCore (Monash University, Australia). Sorted cells were collected in RPMI medium 1640 containing 30% (v/v) fetal bovine serum (FBS) (29).

### 3D TEPC Cultures

Purified TEPCs(~1 × 10^4^) acquired from cell sorting were co-cultured with 2 × 10^5^ irradiated mouse embryonic fibroblasts (MEFs) in 50% growth-factor-reduced Matrigel® (BD Biosciences, U.S.A.), placed into 24-well 0.4 μm transwell inserts (Millipore, Merck, U.S.A.) and incubated with TEC media, as previously described (18). Media was changed every 48 h. Following a seven-day incubation at 37°C in a hypoxic environment (5% O_2_, 10% CO_2_) (30), colony number was determined using an optical (Zeiss Primo Vert, Germany) or multicolor confocal (Leica DMi8, Germany; Monash Micro Imaging) microscope. Colony forming efficiency (CFE%) was determined by dividing the number of colonies formed by the number of seeded TEPCs × 100%.

### Immunocytochemistry of 3D Cultured TEPC Colonies

Transwell inserts were washed twice with PBS and fixed with 4% (w/v) paraformaldehyde (PFA) in PBS for 20 min at room temperature (RT). Antigen retrieval was performed by submerging inserts in sodium citrate buffer (10 mM sodium citrate, 0.05% Tween 20, pH 6.0 in PBS) for 30 min at 95°C. Samples were then washed with washing buffer (0.1% Triton-X in PBS) for 2 h, and blocked with 1% BSA in washing buffer for another hour at RT. Colonies were stained with primary and secondary antibody cocktails (Table 2) for 2 h each at RT in the dark; samples were washed twice (5 min each) with washing buffer in between primary and secondary steps. Lastly, nuclear staining with DAPI was performed for 15 min, followed by two further washes. Transwell insert membranes were cut with a scalpel and placed on a coverslip. The membrane was gently removed from the stained colonies before application of a glass slide with fluorescence mounting media (Dako®, U.S.A.). Images were acquired using a confocal fluorescence microscope (Nikon Eclipse Ti, U.S.A.; Monash Micro Imaging) and analyzed with Fiji-ImageJ software v.2.0.0.

**Table 2:** Antibodies utilized for immunofluorescent staining— immunocytochemistry.

**Antibody**	**Marker**	**Host/isotype/clone**	**Supplier**
β5t	Cortical	Rabbit IgG	MBL International, U.S.A.
Keratin-14	Medullary TEC	Chicken IgY, Poly9060	Biolegend, U.S.A.

### RT-qPCR

The RNAqueous™ Micro Kit (Ambion, U.S.A.) was used to isolate total RNA as per manufacturer's instructions. Briefly, FACS-purified CD45^−^ thymic stromal cell subsets were lysed immediately after sorting (RNAqueous™ Lysis Solution). The cell lysate was mixed with 100% ethanol, and transferred into a silica-based filter which binds RNA. Total RNA was extracted following column purification, sample elution, and DNase treatment, with its concentration and purity measured using a NanoDrop (Thermofisher, U.S.A.). First-strand cDNA synthesis was achieved using the Superscript III reverse transcriptase kit (Invitrogen, U.S.A.). Total RNA was denatured in conjunction with oligo(dT)_20_ (50 μM) primer and dNTP mix for 5 min at 65°C. Samples were immediately placed on ice for 2 min to allow for primer-RNA adherence. The Superscript III reverse transcriptase master mix was added and the reactions run for 50 min at 50°C. Following inactivation for 5 min at 85°C, RNase H was added for 20 min at 37°C, to remove mRNA whilst leaving cDNA template for subsequent RT-qPCR.

RT-qPCR reactions were performed using SYBR Green Supermix-UDG (Invitrogen, Australia), pre-validated primer sequences (Fst and Bmp4; Qiagen, Germany) and cDNA template, in a Corbett Rotor-Gene 3000 (Corbett Research, Australia). The expression of target genes was then analyzed with Rotor-Gene software version 6.1 (Qiagen, U.S.A), relative to GAPDH using the 2ΔΔCt method.

### Serum Analysis for FSH and LH

Whole blood samples were withdrawn via cardiac puncture with thoracotomy using a 26G needle, and collected in a 1.5 ml microcentrifuge tube (31). This procedure was performed immediately after euthanasia. Blood samples were allowed to clot for at least 30 min at RT, then centrifuged at 1,000–2,000 g for 10 min at 4°C. The supernatant (blood sera) was transferred into a new microcentrifuge tube and stored at −80°C. Follicular stimulating hormone (FSH) and luteinizing hormone (LH) levels were later examined via radioimmunoassay.

### Statistical Analysis

All data were analyzed using GraphPad Prism v7.0 software. Independent One- or Two-Way ANOVA tests were run, with the appropriate *post-hoc t*-test performed for parametric tests. A *p*-value of < 0.05 was considered statistically significant. Results are presented as mean + SEM, unless otherwise specified.

## Results

### Phenotypic Differences in Female and Male TEC Subsets With Aging

**Figure 1:**
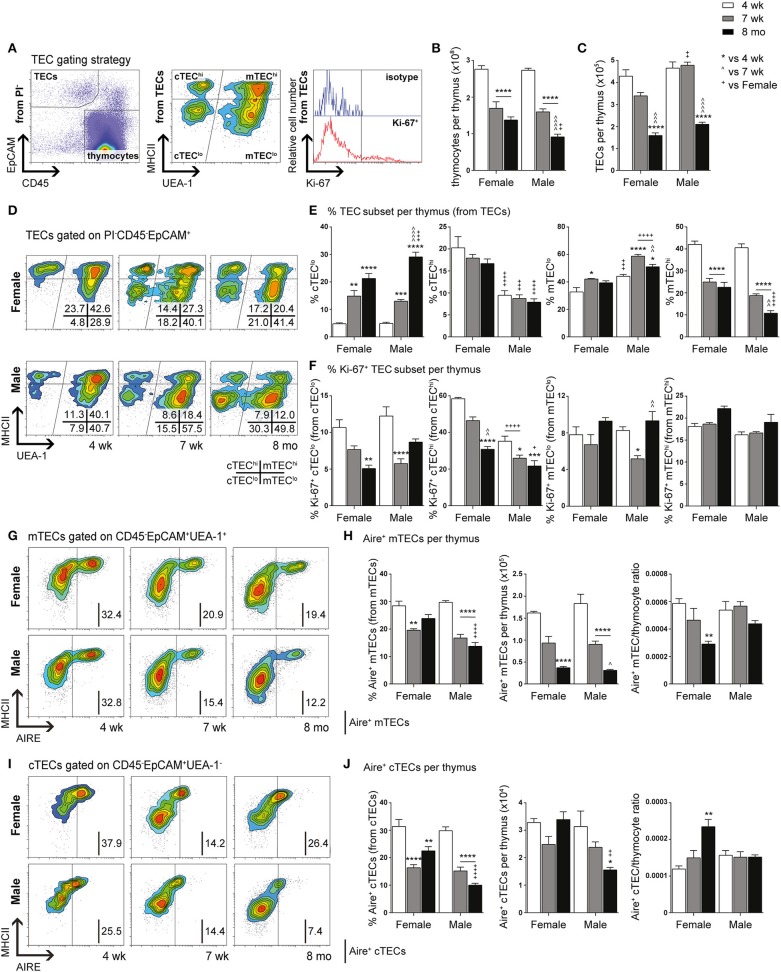
Phenotypic analysis of TEC subsets in female and male mice during aging. **(A)** Gating strategy identifying thymocytes and TECs in 4-week-, 7-week-, and 8-month-old female and male C57BL/6J mice. UEA-1 and MHCII were used to further segregate TECs into cTEC^lo^, cTEC^hi^, mTEC^lo^, and mTEC^hi^ subpopulations. Proliferative cells were identified based on fluorescence exceeding Ki-67 isotype levels. **(B)** Total thymocyte cellularity. **(C)** Total TEC cellularity. **(D)** Representative contour plots depicting proportional changes in TEC subsets in relation to gender with aging. **(E)** Proportion of cTEC^lo^, cTEC^hi^, mTEC^lo^, and mTEC^hi^ subpopulations. **(F)** Proportion of Ki-67^+^ cells within TEC subsets. **(G)** Representative contour plots depicting proportional changes in Aire^+^ mTEC subpopulations in relation to gender with aging. **(H)** Proportion and number of Aire^+^ mTECs per thymus, and Aire^+^ mTEC/thymocyte ratio. **(I)** Representative contour plots depicting proportional changes in Aire^+^ cTEC subpopulations in relation to gender with aging. **(J)** Proportion and number of Aire^+^ cTECs per thymus, and Aire^+^ cTEC/thymocyte ratio. Data presented as mean + SEM (*n* ≥ 3). * vs. 4 wk, ^∧^ vs. 7 wk, ^+^ vs. Female (age matched). *p < 0.05, **p < 0.01, ***p < 0.001, ****p < 0.0001, ordinary two-way ANOVA with Tukey's multiple comparisons.

Comparative immunophenotypic analyzes of TEC subpopulations were performed in 4-week (pre-pubertal), 7-week (post-pubertal), and 8-month-old (middle-aged) C57BL/6J mice, to examine for age- and gender-related differences. Using multiparameter flow cytometry, thymocytes (CD45^+^ EpCAM^−^) and TECs (CD45^−^ EpCAM^+^) were divided after gating on viable (PI^−^) cells (Figure 1A). In addition, MHCII was used to broadly divide TECs into TEC^lo^ and mature (TEC^hi^) subpopulations, with cortical (cTEC, UEA-1^−^) and medullary (mTEC, UEA-1^+^) TECs separated by UEA-1 (29). The proliferative capacity of TEC subsets was also assessed using Ki-67.

Total thymocyte and TEC numbers were found to be similar between 4-week-old female and male mice (Figures 1B,C). By 7-weeks, a drastic decline in thymocytes was observed in both females and males, with males demonstrating a further reduction by 8-months in contrast to female thymocytes which did not decrease significantly. Reduction in overall TEC number was not evident until middle-age. However, proportional differences in female and male TEC subsets were evident from 7-weeks of age (Figure 1D).

A recent study has demonstrated the vast numerical underestimation of TEC subsets associated with enzymatic digestion (32); therefore, we assessed the changes in TEC subsets based on proportional alterations. Gender-associated phenotypic divergence was observed in all age groups, with females exhibiting greater overall cTEC^hi^ and mTEC^hi^ proportions compared to males by middle-age (Figure 1E). In contrast, females accumulated less cTEC^lo^ compared to males by 8-months and males maintained a higher proportion of mTEC^lo^ throughout aging. These findings suggest overall better maintenance of TEC differentiation in females during aging and implicate a greater impediment in mTEC^lo^ to mTEC^hi^ differentiation in males compared to females by middle-age.

Analysis of proliferative capacity in TEC subsets revealed a significant reduction in the proportion of Ki-67^+^ cTEC^lo^ by 8-months in females, with a transient decrease observed in males at 7-weeks (Figure 1F). Female cTEC^hi^ presented with substantially more proliferating cells in comparison to males across all ages which, in addition to better maintenance of cTEC^lo^ to cTEC^hi^ differentiation, may contribute to the higher proportion of cTEC^hi^ evident in females. A decline in proliferation was only evident by middle-age rather than 7-weeks found in males. Furthermore, a transient reduction in mTEC^lo^ proliferation was seen in post-pubertal males. No significant changes in Ki-67^+^ mTEC^hi^ proportion were found with age for both genders. Collectively, these findings indicate gender-specific proliferative patterns that may be impacted by the differential influences of androgens and estrogens.

Since it has been well established that medullary expression of Aire is essential for robust central tolerance [reviewed in (14)], we therefore investigated the impact of aging and gender on Aire^+^ mTECs (Figures 1G,H). A proportional and numerical reduction was evident between 4- and 7-weeks of age in female and male mice, albeit not significant numerically in females until 8-months. By middle-age, females were found to have a significantly higher proportion of Aire^+^ mTECs than males despite the absence of numerical differences. Given the latter observation, it is clear that there is a reduced Aire^+^ mTEC/thymocyte ratio in middle-aged female mice, which may potentially contribute to reduced efficiency of self-tolerance mechanisms. Since a small population of cTEC^hi^ marked by Ly51 and β5t but not K14 or UEA1, expresses intermediate levels of Aire (33), we also analyzed Aire^+^ cTECs (Figures 1I,J), and revealed a similar proportional decline as Aire^+^ mTECs with age and gender, however, no numerical reduction in females was observed. The reduction in male Aire^+^ cTEC number was only significant at 8-months. The concurrent increase in Aire^+^ cTEC/thymocyte ratio of middle-aged female mice with decrease in Aire^+^ mTEC/thymocyte ratio suggests a relationship may exist between these Aire^+^ TEC subpopulations.

### Gender Differences in TEPC Maintenance and Differentiation With Aging

**Figure 2:**
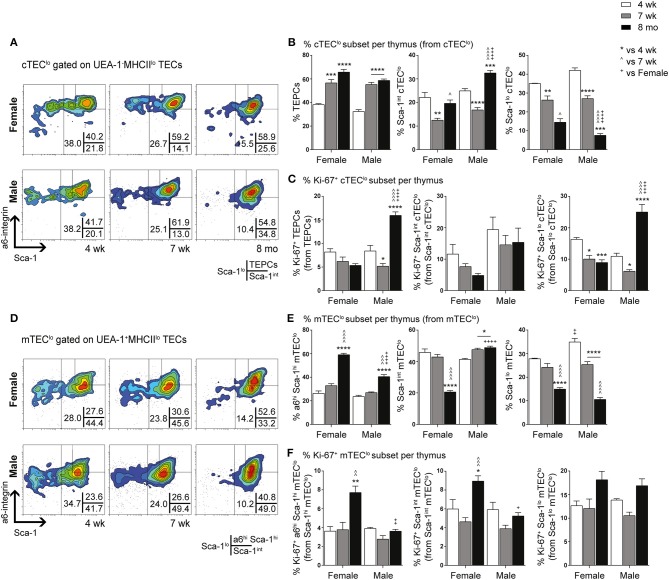
Phenotypic analysis of TEPC and mTEC progenitor subsets in female and male mice during aging. **(A)** Representative contour plots depicting proportional changes in cTEC^lo^ subsets in relation to gender with aging. Antibodies against α6-integrin and Sca-1 were used to segregate cTEC^lo^ into TEPC (α6^hi^ Sca-1^hi^), Sca-1^int^ cTEC^lo^, and Sca-1^lo^ cTEC^lo^ subpopulations. **(B)** Proportion of TEPC, Sca-1^int^ cTEC^lo^, and Sca-1^lo^ cTEC^lo^. **(C)** Proportion of Ki-67^+^ cells within cTEC^lo^ subsets. **(D)** Representative contour plots depicting proportional changes in mTEC^lo^ subsets in relation to gender with aging. Antibodies against α6-integrin and Sca-1 were used to segregate mTEC^lo^ into α6^hi^ Sca-1^hi^ mTEC^lo^, Sca-1^int^ mTEC^lo^, and Sca-1^lo^ mTEC^lo^ subpopulations. **(E)** Proportion of α6^hi^ Sca-1^hi^ mTEC^lo^, Sca-1^int^ mTEC^lo^, and Sca-1^lo^ mTEC^lo^. **(F)** Proportion of Ki-67^+^ cells within mTEC^lo^ subsets. Data presented as mean + SEM (*n* ≥ 3). * vs. 4 wk, ^∧^ vs. 7 wk, ^+^ vs. Female (age matched). *p < 0.05, **p < 0.01, ***p < 0.001, ****p < 0.0001, ordinary two-way ANOVA with Tukey's multiple comparisons.

We have previously demonstrated the existence and function of bipotent postnatal TEPCs in the cTEC^lo^ compartment (10, 18). Given the gender-based differences in cTEC^lo^ and other TEC subsets, we conducted in-depth analyzes of the progenitors within, based on Sca-1 and α6-integrin (α6) expression. cTEC^lo^ was delineated into α6^hi^ Sca-1^hi^ (TEPCs), Sca-1^int^ and Sca-1^lo^ groups, with reduced Sca-1 expression associated with differentiation into single lineage cTEC precursors that generate the Sca-1^lo^ cTEC^hi^ subset (10). FACS analyzes demonstrated similar trends between female and male mice with aging (Figures 2A,B). The proportion of TEPCs increased significantly between 4-weeks and 7-weeks of age in both females and males, with no further change by 8-months. Sca-1^int^ cTEC^lo^ proportions were instead transiently reduced at 7-weeks, with an accumulation in males by 8-months that surpassed females. In contrast, Sca-1^lo^ cTEC^lo^ progressively decreased with age in both genders, with higher proportions observed in middle-aged females than males, suggesting continued TEPC differentiation through Sca-1^int^ in females. Our proliferative studies revealed an increase in the proportion of Ki-67^+^ TEPCs in males beyond female levels by 8-months (Figure 2C), which may partially explain the higher proportion of cTEC^lo^ observed with aging in males. No significant changes in female Ki-67^+^ TEPC proportions were found. Ki-67^+^Sca-1^int^ cTEC^lo^ proportions also remained unchanged with age. Whilst Ki-67^+^ Sca-1^lo^ cTEC^lo^ was found to decrease by 7-weeks in both genders, middle-aged male proportions later increased to levels that exceeded their female counterparts. Together, these results suggest an accumulation of TEPCs in mice, with gender disparity evident in the levels of proliferation of Sca-1^lo^ cTEC^lo^ progenitors for maintenance of the cTEC^hi^ lineage.

Delineation of the mTEC^lo^ subset into α6^hi^ Sca-1^hi^, Sca-1^int^, and Sca-1^lo^ groups revealed similar proportional trends to the cTEC^lo^ compartment during aging (Figures 2D,E). We have previously proposed that the α6^hi^ Sca-1^hi^ TEPC population differentiates toward either the mature Sca-1^lo^ cTEC^hi^ phenotype, or intoα6^hi^ Sca-1^hi^ mTEC^lo^ single lineage precursors that eventually give rise to Sca-1^lo^ mTEC^hi^. The increase in α6^hi^ Sca-1^hi^ mTEC^lo^ by 8-months of age was more evident in females, in parallel to a decrease in Sca-1^int^ mTEC^lo^. Sca-1^lo^ mTEC^lo^ also decreased with age by 8-months in females, but occurred progressively from 7-weeks in males. Proliferative studies revealed an increase in the proportion of Ki-67^+^ α6^hi^ Sca-1^hi^ and Sca-1^int^ mTEC^lo^ in females by 8-months (Figure 2F), with the latter potentially acting to support the maintenance of the Sca-1^int^ mTEC^lo^ subpopulation. The proportion of Ki-67^+^ Sca-1^lo^ mTEC^lo^ did not change significantly during aging in either gender. These data collectively implicate an association between medullary precursors and gender disparity, with reduced differentiation of Sca-1^hi^ to Sca-1^lo^ mTEC^lo^ underpinning the loss of Sca-1^lo^mTEC^hi^ during aging.

### Diminution of TEPC Self-Renewal Capacity Following Puberty

**Figure 3:**
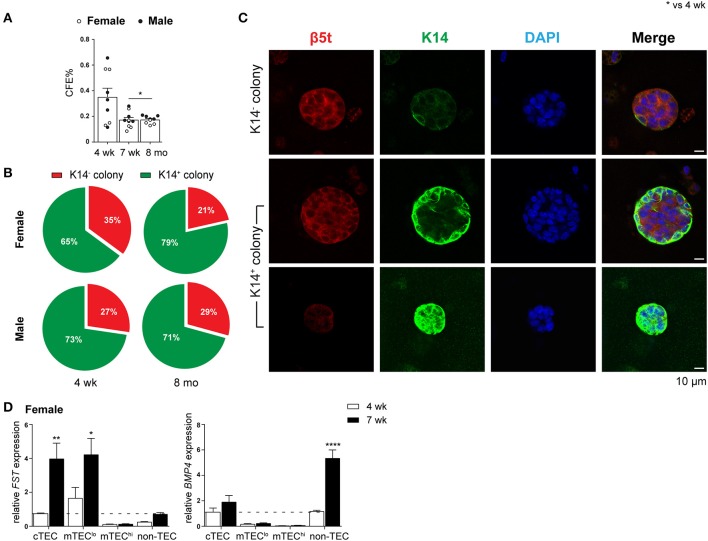
Evaluation of self-renewal and bipotency in female and male TEPC with aging using *in vitro* 3D co-cultures. **(A)** Colony forming efficiency (CFE%) of 4-week-, 7-week-, and 8-month-old TEPCs at D7. Each symbol represents one individual well. **(B)** Proportion of K14^−^ and K14^+^colonies. **(C)** Representative immunofluorescent images of D7 colonies from 4-week-old female TEPC co-cultures. Cortical (β5t; red) and medullary regions (K14; green) from K14^−^ and K14^+^ colonies are shown, together with nuclear staining (DAPI; blue). Scale bar = 10 μm. **(D)** Relative Fst and Bmp4 expression in cTEC, mTEC^lo^, mTEC^hi^, and non-TEC subsets from 4-week- and 7-week-old female mice; normalized to 4-week-old cTEC. Data presented as mean + SEM (*n* ≥ 3). * vs. 4 wk. *p < 0.05, **p < 0.01, ****p < 0.0001, ordinary one- or two-way ANOVA with Tukey's multiple comparisons.

The ability of purified TEPC to self-renew and differentiate into both cTEC and mTEC lineages was investigated through a previously described *in vitro* 3D clonogenic assay (18). The equivalent Sca-1^hi^ population in mTEC^lo^ has been shown to have no bipotent or clonogenic capacity (17, 18). We assessed the CFE% of female and male TEPCs to determine if their ability to self-renew was lost with age (Figure 3A). Following 3D co-culture of purified TEPCs with MEFs for seven days, we found a decline in CFE% from 4- to 7-week-old TEPCs, with no further reduction thereafter. This suggests attenuation of TEPC function from the onset of puberty in both genders. Gender disparity was not observed, possibly due to TEPC exclusion from their native *in vivo* environment. Immunocytochemistry was also performed on colonies generated from 4-week and 8-month old female and male purified TEPCs (Figures 3B,C), to confirm progenitor bipotency and potential age- and gender-associated differences. Colonies were stained with β5t, K14, and DAPI, to identify cortical, medullary and nuclear regions, respectively (10). Although a higher proportion of K14^−^ colonies was observed in 4-week-old female TEPC cultures in comparison to males, no notable difference was seen with age in relation to the prevalence of colonies exhibiting K14 staining (Figure 3B). Several colonies with heterogeneous phenotypes were identified, which were segregated into K14^−^ (β5t^+^K14^−^; cTEC), and differentiating K14^+^ (β5t^+/−^K14^+^) groups (Figure 3C). These findings further validate that the α6^hi^ Sca-1^hi^ cTEC^lo^ population contains bipotent TEPCs in both genders.

Alterations in the Fst-activinA-Bmp4 axis has previously been shown to be one of the mechanisms underlying thymic involution following puberty in male mice (10). Specifically, an increase in TEC expression of Fst, a potent antagonist of activin A, and non-TEC expression of Bmp4 was evident soon after the onset of puberty. These findings prompted us to investigate whether a similar mechanism occurred in females. Comparison of Fst and Bmp4 expression by RT-qPCR analyzes between 4-weeks (pre-puberty) and 7-weeks (post-puberty) of age, showed a substantial increase in Fst expression in cTEC and mTEC^lo^ subsets, and a 4-fold increase in Bmp4 expression by non-TECs (Figure 3D). These results implicate a similar role of TGF-β superfamily molecules in females and males, which is associated with the loss of TEC differentiation following the onset of puberty.

### Gender Disparity in Middle-Aged Mice Following LHRH Receptor Antagonist Treatment

The effect of SHD on thymus regeneration in aging male mice has been widely published (10, 12, 19). The majority of these studies utilized either surgical or chemical castration with a LHRH agonist to block sex steroid production. However, a more recent publication used the LHRH receptor antagonist, degarelix, to rapidly reduce testosterone levels without the initial flare associated with agonists (12). Here, we evaluated the effects of degarelix on TEC regeneration in 8-month-old female and male mice. Mice were given a subcutaneous injection of degarelix (78 μg/g) 48 h prior, so that sex steroids reached castrate levels by day 0 (D0), and multiparameter flow cytometry analysis of the thymus performed at several time points thereafter (D4, D7, D10, D14, and D28).

**Figure 4:**
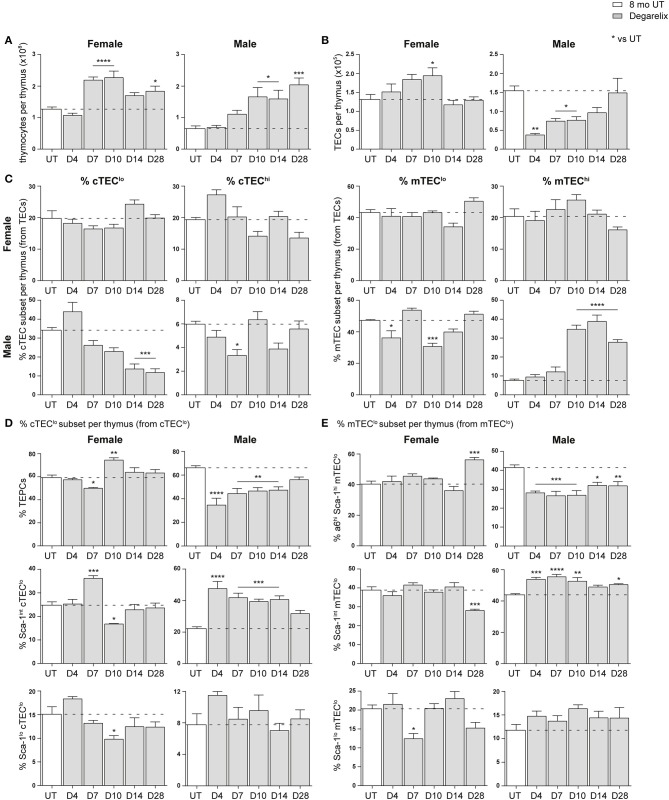
Phenotypic analysis of TEC subsets in middle-aged female and male mice following degarelix treatment. **(A)** Total thymocyte cellularity in 8-month-old female and male mice following degarelix treatment. **(B)** Total TEC cellularity. **(C)** Proportion of cTEC^lo^, cTEC^hi^, mTEC^lo^, and mTEC^hi^ subpopulations. **(D)** Proportion of TEPC, Sca-1^int^ cTEC^lo^, and Sca-1^lo^ cTEC^lo^. **(E)** Proportion of α6^hi^ Sca-1^hi^ mTEC^lo^, Sca-1^int^ mTEC^lo^, and Sca-1^lo^ mTEC^lo^. Data presented as mean + SEM (*n* ≥ 3). * vs. UT. *p < 0.05, **p < 0.01, ***p < 0.001, ****p < 0.0001, ordinary one-way ANOVA with Dunnett's multiple comparisons.

Compared to 8-month old untreated (UT) females, a substantial increase in total thymocyte number was evident at D7, D10, and D28 (Figure 4A). Eight-month old males instead demonstrated a significant increase in thymocyte number from D10 onwards. An ascending, transient trend in TEC number was also evident in females following treatment, with a notable difference at D10 before returning to UT levels (Figure 4B). In contrast, male TEC numbers were significantly reduced, with recovery to UT levels observed only by D14. This initial TEC loss was surprising since degarelix is described as a LHRH receptor antagonist, and suggests some initial LHRH receptor stimulation in males. Although no statistical difference in the proportions of major TEC subsets was seen in females following degarelix treatment (Figure 4C), a gradual decline in male cTEC^lo^ proportions was observed, with mTEC^hi^ proportions surpassing UT levels by D10, following trends seen with surgical castration (10).

We further analyzed the Sca-1^+^ subsets within the cTEC^lo^ population, to investigate whether degarelix treatment triggered TEPC reactivation (Figure 4D). Consistent with reactivation of TEPC, male TEPC proportions were reduced at D4, corresponding with an increase in Sca-1^int^ cTEC^lo^ proportions. This trend was also evident in females, although attenuated and occurred only at D7. Moreover, it aligns with the transient increase in female thymocyte numbers seen at D7 and D10. The Sca-1^lo^ cTEC^lo^ subset did not demonstrate considerable change after degarelix treatment. Analysis of Sca-1^+^ mTEC^lo^ subpopulations revealed a transient reduction in female Sca-1^lo^ mTEC^lo^ at D7, and accumulation of the α6^hi^ Sca-1^hi^ subset at D28 (Figure 4E). Male Sca-1^+^ mTEC^lo^ subpopulation analyzes revealed a similar profile to the Sca-1^+^ subsets within the cTEC^lo^ population, with a reduction of the α6^hi^ Sca-1^hi^ subset from D4 and concurrent increase in Sca-1^int^ mTEC^lo^. Collectively, these findings suggest that although the increase in female thymocyte production occurs in a homeostatic manner with no major changes in TEC subset proportions, male thymopoiesis and mTEC^hi^ generation is instead enhanced following degarelix treatment conceivably via mobilization of both TEPC and mTEC^lo^ progenitor populations.

### SHD-Enhanced Recovery of Thymocytes and TECs Following Cy Damage

The cytoablative effects of chemotherapy on the thymus have been previously investigated in young male mice (5, 34). We report herein, gender-related phenotypic differences in the TEC compartment of middle-aged mice following chemotherapy damage, and examined the extent to which SHD could enhance the kinetics of thymus regeneration in females compared to males.

Eight-month-old mice were chemically castrated with degarelix at D-2, followed by an intraperitoneal injection of cyclophosphamide (Cy) at D-1 and D0 (hereafter referred to as the Cy + Deg treatment group); an identical dose of Cy alone was also administered to a control group (Figure S1A). Thymus cell populations were subsequently analyzed via flow cytometry at D4, D7, D10, D14, and D28 after the last day of Cy injection. Assessment of estrogen and progesterone production was also performed for females at each time point by measuring LH and FSH, respectively (Figure S1B).Testosterone levels have been previously shown to reach castrate levels within 24 h of degarelix treatment (12). The fluctuating LH and FSH levels in UT controls suggest a normal oestrous cycle. As expected, degarelix inhibited the secretion of LH and FSH from the anterior pituitary. Serum LH concentrations were depleted to castration levels until at least D28, with an average of < ~0.166 ng/ml whilst FSH was persistently suppressed at an average of ~1.7 ng/ml across all time points.

**Figure 5:**
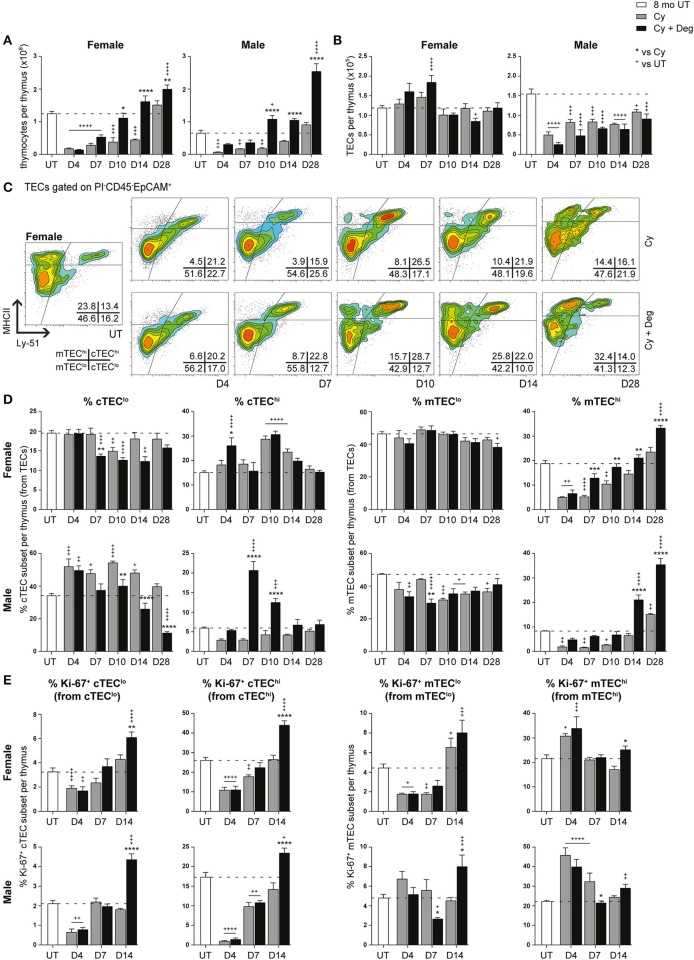
Phenotypic analysis of TEC subsets in middle-aged female and male mice with degarelix treatment following Cy damage. **(A)** Total thymocyte cellularity in 8-month-old female and male mice with or without degarelix post-Cy treatment. **(B)** Total TEC cellularity. **(C)** Representative contour plots depicting proportional changes in female TEC subsets with or without degarelix post-Cy treatment. MHCII and Ly-51 were used to segregate TECs into cTEC^lo^, cTEC^hi^, mTEC^lo^, and mTEC^hi^ subpopulations. **(D)** Proportion of cTEC^lo^, cTEC^hi^, mTEC^lo^, and mTEC^hi^ subpopulations. **(E)** Proportion of Ki-67^+^ cells within TEC subsets. Data presented as mean + SEM (*n* ≥ 3). * vs. Cy, ^+^ vs. UT. *p < 0.05, **p < 0.01, ***p < 0.001, ****p < 0.0001, ordinary two-way ANOVA with Sidak's multiple comparisons.

A dramatic reduction in thymocyte number was evident from D4 in both female and male Cy groups compared to UT controls (Figure 5A). Endogenous regeneration to UT levels in this group was only achieved by D28 in females and earlier in males, which had a lower base number. Cy + Deg treatment enhanced the kinetics of thymocyte recovery, showing statistical significance from D10 and reaching similar levels in both females and males. Interestingly, thymocyte number in males regenerated beyond UT levels as early as D10, whereas females achieved the same result at D28. By D28, thymocyte numbers were increased to ~1.6-fold UT levels in females, and ~3.9-fold UT levels in males. These findings suggest androgens have a more suppressive effect than estrogens on thymopoiesis. Surprisingly, the loss of thymocytes from Cy treatment in females did not coincide with significant differences in total TEC number, and only minor changes were observed with Cy + Deg treatment (Figure 5B). Loss in total TEC number was, however, observed in males following Cy treatment; perhaps related to a greater reliance on proliferation to maintain TEC numbers, with degarelix having no beneficial effect in the regeneration of total TEC number.

Further examination of TEC subsets however, revealed a significant regeneration of mature TEC subsets (Figures 5C,D, Figure S1C). Using Ly-51^+^ expression to define cTECs from mTECs (Ly-51^−^), we found the mTEC^hi^ subset to be the most affected by Cy in both genders, as evidenced by its dramatic proportional loss at D4 (Figure 5D). Complete endogenous recovery of mTEC^hi^ was apparent between D14 and D28. Whilst there was no proportional loss in cTEC^hi^ cells at D4, Cy damage did induce endogenous mobilization of cTEC^lo^ at D10 in females. This was coincident with enhanced cTEC^hi^ proportions at D10 and D14 before returning to UT levels at D28. In contrast, the male cTEC^lo^ subset demonstrated a persistent proportional increase immediately following Cy damage compared to UT levels. Notably, the reduced mTEC^lo^ proportion observed from D10 suggests that, in conjunction with TEPC reactivation, male thymic regeneration may involve a higher degree of mTEC^lo^ differentiation for mTEC^hi^ recovery post-Cy damage compared to females.

Degarelix treatment enhanced mTEC^hi^ recovery post-Cy damage, reaching significance by D7 in females and D14 in males (Figure 5D). Significant cTEC^lo^ mobilization was evident from D7 to D14 in females, returning to UT levels by D28. Cy + Deg treatment in females also prompted an initial wave of cTEC^hi^ expansion at D4. This expansion implicates sequential recovery of cTEC^hi^ and mTEC^hi^, possibly in parallel with the recovery of thymocyte populations. Mobilization of the cTEC^lo^ compartment was also observed in Cy + Deg treated males, which was demonstrated by reduced cTEC^lo^ proportions from D10 compared to Cy alone. Interestingly, Cy + Deg treatment also prompted an initial wave of cTEC^hi^ expansion, albeit delayed at D7-10 compared to females. This event provides insight on the mechanisms behind SHD-induced thymopoiesis. The early reduction in male mTEC^lo^ proportions suggests that overall enhancement of gender-specific endogenous repair accompanies the common thymic regenerative events induced by sex steroid deprivation.

Due to the pronounced changes in male mTEC subpopulations with Cy + Deg treatment, we examined for enhancements in their peripheral T cell pool. Total splenocyte numbers were enhanced with degarelix by D28 (data not shown), albeit no notable differences within splenic subsets were observed. We detected higher peripheral T cell numbers within brachial and inguinal lymph nodes (Figure S2A). This increase was evident in naïve (CD62L^+^ CD44^lo^) CD4^+^ and CD8^+^ T cell subpopulations from D14, with enhancement also seen in central memory (CM, CD62L^+^ CD44^hi^) and effector memory (EM, CD62L^−^ CD44^hi^) cell numbers. Notably, EM cells reached young levels by D14 (data not shown). Ki-67 analyzes indicated involvement of homeostatic expansion of these populations from D10 (Figure S2B). Enhanced T regulatory (Treg, CD4^+^ CD25^+^ Foxp3^+^) cell recovery with degarelix treatment was also detected from D14, with virtual memory (VM) cells (CD8^+^ CD122^hi^ CD44^hi^ CD49d^−^) exhibiting homeostatic expansion in a lymphopenic environment (35) and clearly responding to Cy damage by D10 (Figure S2C). Additionally, bone marrow analyzes showed no enhancement in hematopoietic stem cell (HSC) numbers with degarelix treatment prior to D14, with no alterations in lymphoid-primed multipotent progenitors (LMPPs) and common lymphoid progenitors (CLPs) (data not shown). As the spike in thymocyte numbers occurs at D10, it is likely that the early changes induced by degarelix treatment are due to thymus-intrinsic mechanisms.

Ki-67 expression studies revealed an increase in mTEC^hi^ proliferation at D4 post-Cy damage in both genders (Figure 5E). This finding may reflect an immediate endogenous proliferative response to the dramatic loss of mTEC^hi^ with Cy treatment. Given that Cy + Deg treatment produced a small differential increase in Ki-67^+^ mTEC^hi^ at D14, it is unlikely that the significant regeneration of the mTEC^hi^ subset with degarelix was mostly due to enhanced proliferation of existing cells. For both genders, the cTEC compartments demonstrated significant reductions in Ki-67 expressing cells at D4 due to Cy, with subsequent endogenous recovery enhanced with degarelix treatment to beyond UT levels at D14. Although a similar trend was observed in the mTEC^lo^ subset of females, the male mTEC^lo^ subpopulation instead demonstrated no decline in proliferative capacity at D4 post-Cy damage. This may also reflect an immediate proliferative response in male mTEC^lo^, supporting the increased reliance on existing mTEC^lo^ progenitors to replenish the mTEC^hi^ population at this age, compared to females. This finding further alludes to the differences between female and male TEC maintenance.

### SHD-Enhanced Recovery of Aire^+^ mTEC Following Cy Damage

**Figure 6:**
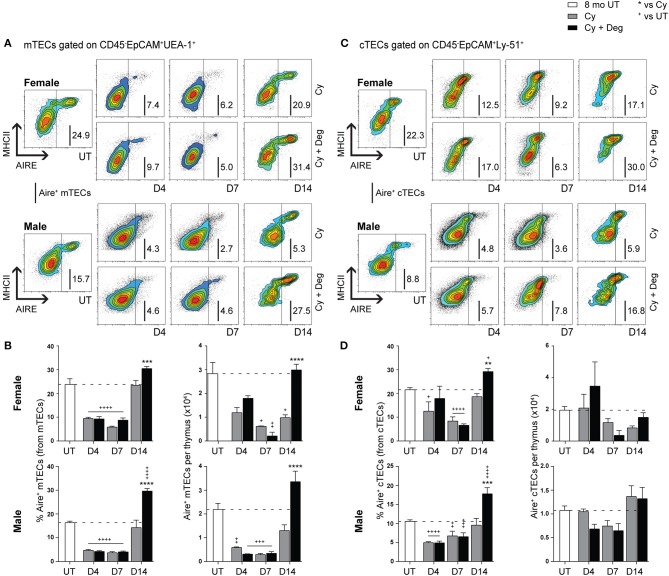
Phenotypic analysis of Aire^+^ mTECs and cTECs in middle-aged female and male mice with degarelix treatment following Cy damage. **(A)** Representative contour plots depicting proportional changes in the Aire^+^ mTEC subpopulation with or without degarelix post-Cy treatment. **(B)** Proportion and number of Aire^+^ mTECs per thymus. **(C)** Representative contour plots depicting proportional changes in the Aire^+^ cTEC subpopulation with or without degarelix post-Cy treatment. **(D)** Proportion and number of Aire^+^ cTECs per thymus. Data presented as mean + SEM (*n* ≥ 3). * vs. Cy, ^+^ vs. UT. *p < 0.05, **p < 0.01, ***p < 0.001, ****p < 0.0001, ordinary two-way ANOVA with Sidak's multiple comparisons.

Due to the enhanced recovery of thymocytes with degarelix treatment post-Cy damage, we examined Aire expression within mTECs to determine whether self-tolerance mechanisms were in place. Our analyzes revealed a loss of Aire^+^ mTEC after Cy, and enhanced recovery with degarelix treatment in both genders (Figures 6A,B). A proportional and numerical loss of Aire^+^ mTEC due to Cy damage was seen, with restoration to UT levels through endogenous repair by D14 (Figure 6B). Treatment with degarelix resulted in similar recovery kinetics in both males and females, however, Aire^+^ mTEC proportions and numbers at D14 were found to be at least 2-fold higher than Cy alone groups. These data suggest maintenance of central tolerance, although further Aire-dependent tissue-restricted antigen (TRA) expression studies would be required to confirm this. Interestingly, no significant numerical changes were observed in Aire^+^ cTECs following Cy and Cy + Deg treatments, despite a proportional increase at D14 in both genders (Figures 6C,D).

### SHD-Enhanced Recovery of TEPCs Following Cy Damage

**Figure 7:**
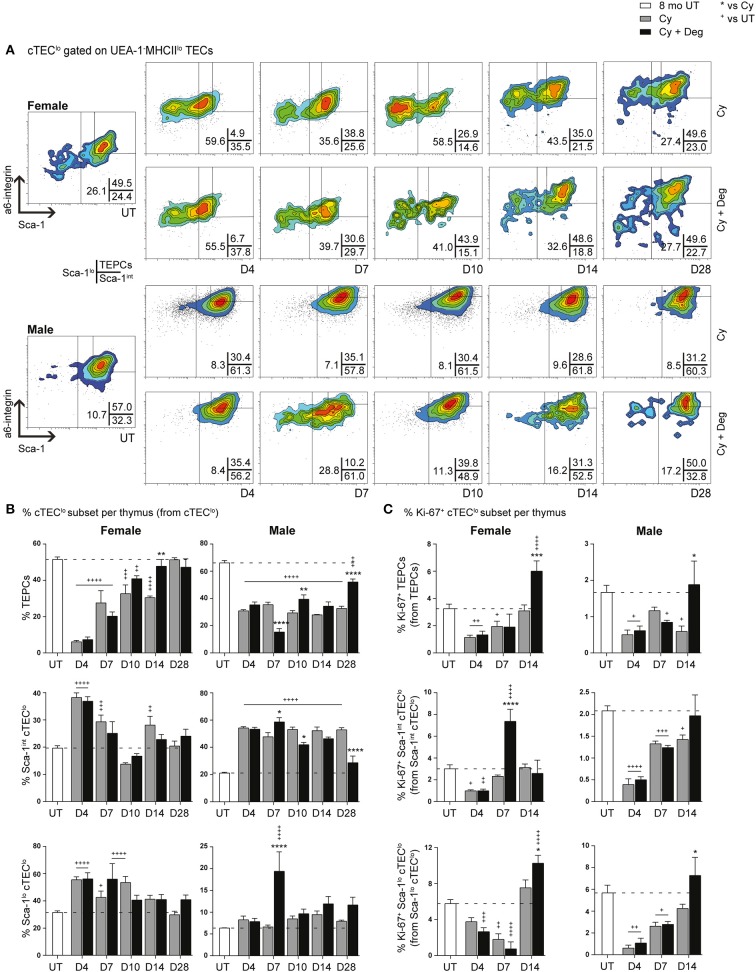
Phenotypic analysis of TEPC subsets in middle-aged female and male mice with degarelix treatment following Cy damage. **(A)** Representative contour plots depicting proportional changes in cTEC^lo^ subsets with or without degarelix post-Cy treatment. Antibodies against α6-integrin and Sca-1 were used to divide cTEC^lo^ into TEPC (α6^hi^ Sca-1^hi^), Sca-1^int^ cTEC^lo^, and Sca-1^lo^ cTEC^lo^ subpopulations. **(B)** Proportion of TEPC, Sca-1^int^ cTEC^lo^, and Sca-1^lo^ cTEC^lo^. **(C)** Proportion of Ki-67^+^ cells within cTEC^lo^ subsets. Data presented as mean + SEM (*n* ≥ 3). * vs. Cy, ^+^ vs. UT. *p < 0.05, **p < 0.01, ***p < 0.001, ****p < 0.0001, ordinary two-way ANOVA with Sidak's multiple comparisons.

Given the absence of pronounced proliferative changes in the mTEC^hi^ subset with degarelix-induced regeneration, we proposed that the increase in mTEC^hi^ originated from differentiation of progenitor cell populations; therefore, we investigated proportional changes in the cTEC^lo^ subset where bipotent TEPCs reside (Figures 7A,B). A substantial reduction of TEPCs was evident at D4 for Cy and Cy + Deg treated females and males compared to UT controls (Figure 7B), with concurrent significant increases in Sca-1^int^ cTEC^lo^ and Sca-1^lo^ cTEC^lo^ proportions. These trends suggest an early endogenous response to Cy-induced TEC loss through immediate TEPC mobilization, but does not rule out direct damage to TEPCs. Thereafter, gradual TEPC renewal was evident in both treatment groups and genders, with a higher TEPC proportion observed in Cy + Deg at D14 in females and D10 in males when compared to Cy alone controls. TEPC homeostasis returned in females by D28, however males did not achieve this result even with degarelix treatment. Interestingly, degarelix treatment appeared to induce a transient reduction in male TEPC proportion at D7, which occurred simultaneously with an increase in Sca-1^lo^ cTEC^lo^ proportion; a dramatic surge in cTEC^hi^ was also observed at this time (Figure 5D). This finding supports our proposal of sequential TEC recovery of cTEC^hi^ and mTEC^hi^. Moreover, the enhanced TEPC renewal with degarelix treatment from D14 in females appears to be associated with proliferation, as evidenced by increased Ki-67 expression (Figure 7C). Transient proliferation in Cy + Deg females was also evident in Sca-1^int^ cTEC^lo^ and Sca-1^lo^ cTEC^lo^ populations at D7 and D14, respectively. No transient enhancement in proliferation was observed in these subsets in Cy + Deg males. From these data, we propose that Cy damage induces immediate endogenous TEPC reactivation and differentiation in middle-aged mice, with degarelix enhancing TEPC renewal at D14 by promoting proliferation.

### SHD-Enhanced Differentiation and Proliferation Within mTEC^lo^ Subsets Following Cy Damage

**Figure 8:**
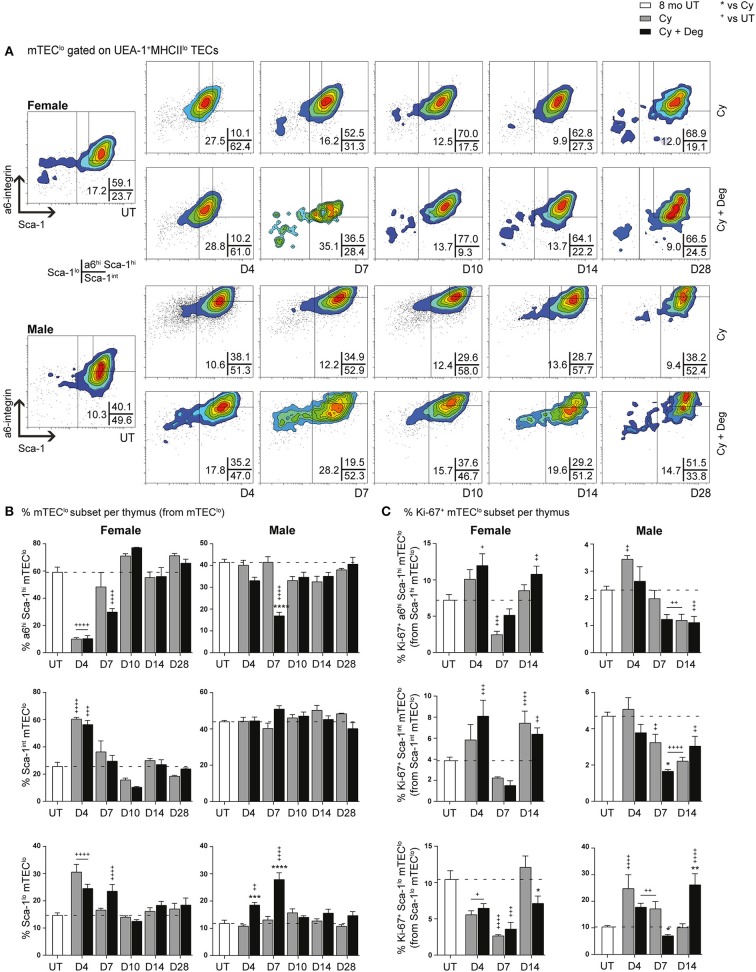
Phenotypic analysis of mTEC progenitor subsets in middle-aged female and male mice with degarelix treatment following Cy damage. **(A)** Representative contour plots depicting proportional changes in mTEC^lo^ subsets with or without degarelix post-Cy treatment. Antibodies against α6-integrin and Sca-1 were used to segregate mTEC^lo^ into α6^hi^ Sca-1^hi^mTEC^lo^, Sca-1^int^ mTEC^lo^, and Sca-1^lo^ mTEC^lo^ subpopulations. **(B)** Proportion of α6^hi^ Sca-1^hi^ mTEC^lo^, Sca-1^int^ mTEC^lo^, and Sca-1^lo^ mTEC^lo^. **(C)** Proportion of Ki-67^+^ cells within mTEC^lo^ subsets. Data presented as mean + SEM (*n* ≥ 3). * vs. Cy, ^+^ vs. UT. *p < 0.05, **p < 0.01, ***p < 0.001, ****p < 0.0001, ordinary two-way ANOVA with Sidak's multiple comparisons.

Since single lineage medullary precursors originating from TEPCs give rise to mTEC^hi^, we investigated for shifts within the mTEC^lo^ subset (Figures 8A,B). In both groups, a substantial reduction of a6^hi^ Sca-1^hi^ mTEC^lo^ in parallel with an increase in Sca-1^int^ mTEC^lo^ and Sca-1^lo^ mTEC^lo^ populations was evident for females at D4, suggesting similar mobilization events observed with cTEC^lo^ populations were involved in endogenous recovery (Figure 8B). Although this trend was absent in males, a transient increase in Sca-1^lo^ mTEC^lo^ proportion was however detected at D4 and D7 in degarelix groups, with a reduced α6^hi^ Sca-1^hi^ mTEC^lo^ proportion seen at D7. These data suggest a gender-disparate mobilization of α6^hi^ Sca-1^hi^ mTEC^lo^, which may explain the accelerated recovery of the mTEC^hi^ subset in Cy + Deg treated females in terms of proportional increases in mTEC^hi^, reaching 20% by D7–10 in females but males not achieving this level until D14. Our proliferative analyzes showed a loss of Ki-67^+^ Sca-1^lo^ mTEC^lo^ at D4 following Cy treatment in females, however the equivalent population had increased in males, supporting immediate endogenous proliferation of existing Sca-1^lo^ mTEC^lo^ precursors for mTEC^hi^ regeneration (Figure 8C). This was enhanced at D14 with degarelix treatment which may relate to replenishment of this population. Together, our data implicates differentiation, rather than proliferation, as the driving force of endogenous medullary repair in females, with males engaging both mechanisms for mTEC^hi^ recovery.

## Discussion

Progressive physiological impediment of naïve T cell generation can result in severe clinical complications following chemotherapy treatment. These conditions increase the incidence of opportunistic infections, which result in increased patient morbidity and mortality. Hence, a clear need for immune reconstitution strategies is required. Here, we examined one promising approach to restore aged-thymic function—the administration of LHRH-analogs. Given the lack of research conducted in females, we initially investigated the mechanisms behind age-related thymic involution in relation to murine sexual dimorphism. As our findings indicated phenotypic differences between females and males, we subsequently examined the impact of LHRH receptor antagonist, degarelix, on thymic recovery following Cy damage in middle-age mice, to determine whether gender disparity was apparent.

Through flow cytometric analyzes, we performed a comparative phenotypic assessment of thymic cell subpopulations in pre-pubertal, post-pubertal, and middle-aged female and male mice. We found the age-related decline in thymocyte number to be more pronounced in males, and suggest that this disparity may result from estrogen- (36) and androgen-specific mechanisms of action (37), which impact on thymocytes and/or thymic stromal cell populations. Despite greater proportions of cTEC^hi^ and mTEC^hi^ subpopulations in females at middle-age, which suggests better maintenance of TEC differentiation compared to males, a female-exclusive reduction in Aire^+^ mTEC/thymocyte ratio was observed. Their lower Aire^+^ mTEC/thymocyte ratio may be associated with an imbalance in central tolerance or Treg cell development that may contribute to the increased clinical risk of autoimmunity seen in female patients (38). Together with the age-associated increase in female Aire^+^ cTEC/thymocyte ratio, we propose that there is an impediment in Aire^+^ mTEC^hi^ differentiation, and that Aire^+^ cTEC^hi^ may be its upstream precursor. Although localized at the cortico-medullary junction (33), which allows for prompt transition into the medulla following differentiation, the Aire^+^ cTEC^hi^ population has previously been shown through ectopic studies to be incapable of ameliorating autoimmune pathology alone, and does not induce TRA genes (39). As these ectopic studies utilized β5t/Aire-transgenic mice that exclusively express Aire in the cortex, further research is required to determine whether Aire^+^ cTEC^hi^ are quiescent upstream precursors of Aire^+^ mTEC^hi^. Their association with receptor activator of nuclear factor κ B (RANK) signaling, which has been demonstrated to regulate Aire expression in mTECs (40, 41), also warrants investigation.

Our data reveal greater impairment in male TEC^lo^ to TEC^hi^ differentiation with aging than females. The pronounced increase in male mTEC^lo^ proportion following puberty, together with a reduction in Ki-67, suggests that there is a gender-related block in mTEC^lo^ to mTEC^hi^ differentiation. Moreover, the proportion of male cTEC^lo^ significantly exceeds that of females by middle-age, hinting at more severe impediment in TEPC differentiation. A potential underlying mechanism for these events was recently reported (10), and relates to a 6-fold increase in male cTEC and mTEC^lo^ Fst expression post-puberty, and its antagonistic relationship to activin A and Bmp4 signaling. Following our assessment of these TGF-β superfamily members in pre- and post-pubertal females, we revealed a 4-fold increase in female cTEC and mTEC^lo^ subsets, which suggests a similar but less profound role to males in the impediment of differentiation through inhibition of activin A signaling. The post-pubertal increase in Bmp4 expression by supporting stromal cells was, however, greater in females, and implicates better progenitor maintenance. Its receptor, Bmpr2, is primarily expressed on cTEC^lo^ and mTEC^lo^ progenitors (42), and was proposed to have a role in maintaining progenitor populations at the expense of differentiation (10). However, Bmp4 induced self-renewal of progenitors may also have a role in thymus regeneration following damage, as demonstrated following irradiation (43).

We have previously proposed that the bipotent α6^hi^ Sca-1^hi^ TEPC population differentiates toward a single lineage cTEC precursor expressing low levels of Sca-1, which in turn differentiates into the mature Sca-1^lo^ cTEC^hi^ phenotype, as well as into Sca-1^hi^ mTEC^lo^ single lineage precursors (10). Despite no obvious differences in TEPC colony phenotype and CFE with gender, our *in vitro* 3D co-culture studies suggest that there is an immediate impairment in self-renewal capacity following puberty. This finding somewhat correlates with a previous study that demonstrated reduced TEC CFE with aging (44), albeit no immediate post-pubertal attenuation was observed. As the interpretation of *in vitro* results is influenced by elements within the culture systems themselves, in conjunction with the lack of sex steroids in such systems, supporting *in vivo* studies will be required to truly elucidate whether gender differences in TEPC function exist throughout aging. Nonetheless, our phenotypic assessments identified an accumulation of TEPCs following puberty in both males and females, which presented with a reduction in downstream Sca-1^lo^ cTEC^lo^ that was more pronounced in males by middle-age. Given the increased proliferation of Sca-1^lo^ cTEC^lo^ in middle-aged males, and the accumulation of Sca-1^int^ cTEC^lo^, we suggest that there may be a block in male Sca-1^int^ cTEC^lo^ differentiation. Hence, these data indicate better maintenance of TEPC function in middle-aged females. Analyzes conducted with the assumption that our α6^hi^ Sca-1^hi^ phenotype can also identify mTEC^lo^ precursors revealed gender disparity in relation to the maintenance of these immature medullary subpopulations by middle-age, yet no differences between female and male Sca-1^lo^ mTEC^lo^ proportions were observed. Not surprisingly, α6^hi^ Sca-1^hi^ mTEC^lo^ do not fall within the mTEC-II phenotype which express Aire and other mature mTEC markers (45). Further research is, however, required to establish the link between α6^hi^ Sca-1^hi^ mTEC^lo^ and previously identified postnatal mTEC progenitor phenotypes such as stage-specific embryonic antigen-1 (SSEA-1)^+^ Claudin (Cld)3,4^hi^ TECs (46, 47).

Given that gender disparity was observed in our aging studies, we compared the regenerative effect of LHRH receptor-antagonist, degarelix, on the middle-aged female thymus to their male counterparts. Transient reversal of thymic involution was observed in females between D7 and D10 post-degarelix treatment, with negligible changes in TEC subset proportions suggesting progenitor activation followed by homeostatic TEC maintenance. This theory is further supported by the decreased proportion of TEPCs at D7 and subsequent increase by D10, which implicates brief progenitor reactivation that coincided with the expansion of thymocytes. Conversely, degarelix treatment of middle-aged males resulted in delayed thymic regeneration, but expansion of thymocyte cellularity was sustained until at least D28. The pronounced, progressive reduction in cTEC^lo^, in conjunction with increased mTEC^hi^ from D10, suggest persistent differentiation into mature TEC subsets which is exclusive to males. Decreased TEPC and α6^hi^ Sca-1^hi^ mTEC^lo^ proportions until at least D14 further supports this notion. Together, these findings implicate degarelix as having a more prominent effect on male TEC subsets and/or that androgens may be more detrimental to T cell generation than estrogens. While alterations in the mobilization of bone marrow precursors can also partially explain our observations (48), our peripheral organ analyzes on middle-aged male mice did not identify an early enhancement in the provision of BM precursors with degarelix treatment (data not shown). Hence, these events are likely to be thymus-intrinsic.

Degarelix treatment was found to enhance the kinetics of thymocyte recovery in Cy-treated middle-aged mice, albeit no gender-based differences and no significant improvement in TEC number were observed. Thymocyte numbers surpassed untreated levels in females by D28 and D10 in males, indicating that androgens may have a more suppressive effect than estrogens on thymopoiesis. This enhancement in thymocyte recovery was also reported in a recent study (12), which demonstrated return to untreated levels by D42 in middle-aged male mice following irradiation. Although there is a lack of data regarding degarelix treatment following irradiation prior to this time point, it is likely that an association exists between the kinetics of thymocyte recovery and the type of cytoablative injury inflicted. Such differences are bound to have clinical implications, and warrant further investigation. Interestingly, total TEC number was exclusively reduced in male animals after Cy treatment. This reduction did not recover back to UT levels by D28, and hints at the possibility of an imbalance in Aire^+^ mTEC/thymocyte ratio, and hence an impediment in central tolerance. The ratio at D14 in males (data not shown) revealed that this was unlikely the case in degarelix treated mice, with early recovery of mTEC^hi^ proportions observed. Our TEC subset analyzes demonstrated transient cTEC^lo^ mobilization with degarelix in females at D7, while mobilization in males was detected from D10. These trends were consistent with degarelix alone data. The enhanced, yet transient, recovery of cTEC^hi^ observed at D4 in females, and D7–10 in males, implicate faster kinetics of recovery in the former. Moreover, both males and females demonstrated improved mTEC^hi^ recovery. This augmentation was detected earlier in females, which further supports their better recovery kinetics. Since the alterations in mTEC subpopulations were more pronounced in males with Cy + Deg treatment, we conducted analyzes with regards to their peripheral T cell pool. Enhanced T cell recovery was observed in lymph nodes, resulting predominantly from proliferation. This enhancement is consistent with previous reports (49, 50), which also demonstrated an absence of immunosuppressive effects with LHRH receptor-antagonist treatment. Increased Treg and VM cell subpopulations also contributed to early peripheral immune reconstitution (51, 52).

Through our proliferative studies, it appeared that degarelix contributed to thymic repair predominantly by augmenting cTEC proliferation by D14, with males demonstrating a more pronounced proportional increase in Ki-67^+^ cTEC^lo^ than females. This disparity likely relates to the persistent mobilization of cTEC^lo^ in middle-aged males, which would in turn require replenishment. The increased proportion of mTEC^hi^ was likely a consequence of progenitor differentiation, rather than proliferation. These data are supported by previous findings, which showed that adult mTEC maintenance and regeneration occurs through β5t^−^ lineage-restricted cells (13). Whether alterations in medullary stromal signaling to the cortex contribute vastly to this process (53) is yet to be investigated. Examination of TEPCs revealed a prominent difference between males and females treated with degarelix after Cy damage. Whilst degarelix enhanced TEPC recovery in females to UT levels by D14, recovery of the progenitor pool was not achieved in males despite enhanced TEPC proliferation at D14 in both genders. This may be due to continued mTEC^hi^ production beyond D28, or possibly indicate TEPC senescence. Analyzes at later time points will be required to determine whether male TEPCs eventually return to UT levels. Furthermore, the absence of decline in male α6^hi^ Sca-1^hi^ mTEC^lo^ at D4 implicates gender disparity in the maintenance of medullary progenitors, which would subsequently impact on the recovery of the mTEC^hi^ subset.

This study details, for the first time, the relationship between sexual dimorphism and TEC aging from the pre-pubertal stages of life to middle-age. We revealed a potential imbalance in central tolerance that may explain the increased incidence of autoimmunity in middle-aged females. A higher Sca-1^lo^ cTEC^lo^ proportion was, however, seen in females, which appears to be associated with better maintenance of differentiation compared to males in this age group. We also demonstrated that degarelix was more effective for thymic regeneration in middle-aged males, and possibly relies on both progenitor reactivation and proliferation. Furthermore, the enhanced recovery of TEC^hi^ subsets in females treated with degarelix and Cy precedes the recovery in males and implicates faster kinetics of recovery. This likely relates to better maintenance of progenitor function in middle-aged females. Taken together, these findings stress the relevance of sexual dimorphism in adaptive immunity, and suggest a plausible benefit to analyzing naïve T cell output in prostate and breast cancer patients treated with LHRH-analogs. Investigations into the potential negative impact of multiple-dose chemotherapy on the SHD-reactivated thymus are also required, to ascertain whether alterations in cancer treatment protocols could be beneficial.

## Data Availability Statement

The datasets generated for this study are available on request to the corresponding author.

## Ethics Statement

The animal study was reviewed and approved by the Monash University Animal Ethics Committee.

## Author Contributions

AC conceptualized and designed the study and analyzed/interpreted data. MH, KW, JG, AA, and KQ performed experiments and/or data analysis. AC, MH, KW, JG, and AA contributed to drafting the original manuscript. All authors critically evaluated the manuscript.

### Conflict of Interest

The authors declare that the research was conducted in the absence of any commercial or financial relationships that could be construed as a potential conflict of interest.
