# Supplementary material for: Gender Disparity Impacts on Thymus Aging and LHRH Receptor Antagonist-Induced Thymic Reconstitution Following Chemotherapeutic Damage
Source: Front Immunol. 2020 Mar 3;11:302. doi: 10.3389/fimmu.2020.00302 (PMC7062683; doi:10.3389/fimmu.2020.00302)
Supplement: Supplementary file 1 [file Data_Sheet_1.docx]

Supplementary Material

# Supplementary Figures


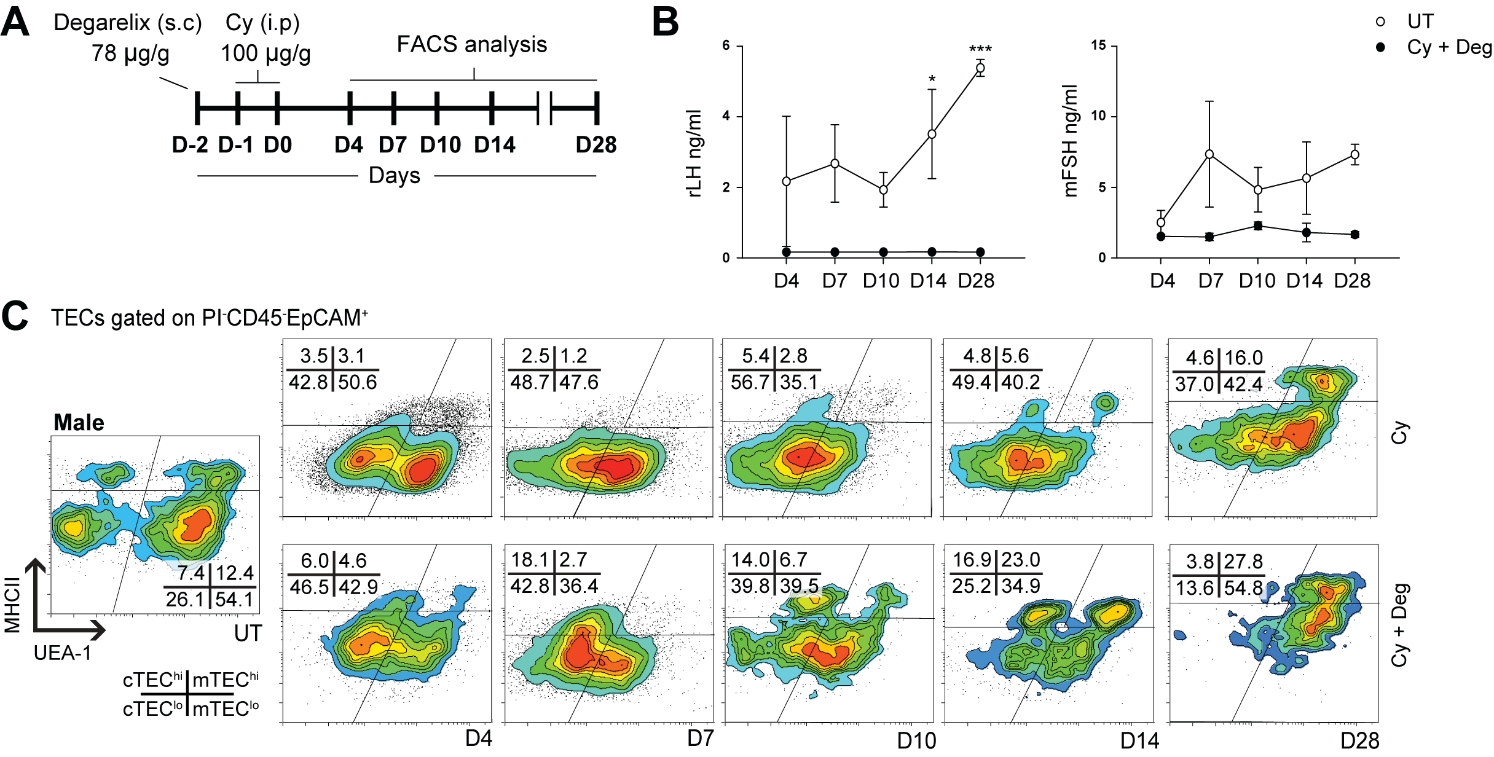


**Supplementary Figure 1.** Degarelix treatment in middle-aged mice following cyclophosphamide damage. **(A)** Schematic illustrating the protocol utilised for degarelix treatment of middle-aged mice. For all experiments, D0 was designated as the day of completion of treatment. Mice were injected with degarelix subcutaneously 48 hours prior (D-2) to allow time for estrogen production to reach castrate levels by D0. Cyclophosphamide was injected intraperitoneally on two consecutive days (D-1 and D0) to simulate its clinical application. **(B)** Serum LH and FSH concentrations in 8-month-old female mice following degarelix treatment. **(C)** Representative contour plots depicting proportional changes in male TEC subsets with or without degarelix post-cyclophosphamide treatment. UEA-1 and MHCII were used to segregate TECs into cTEC^lo^, cTEC^hi^, mTEC^lo^, and mTEC^hi^ subpopulations. Data presented as mean ± SEM (n>3). * vs UT. *p<0.05, ***p<0.001, ordinary two-way ANOVA with Sidak’s multiple comparisons.


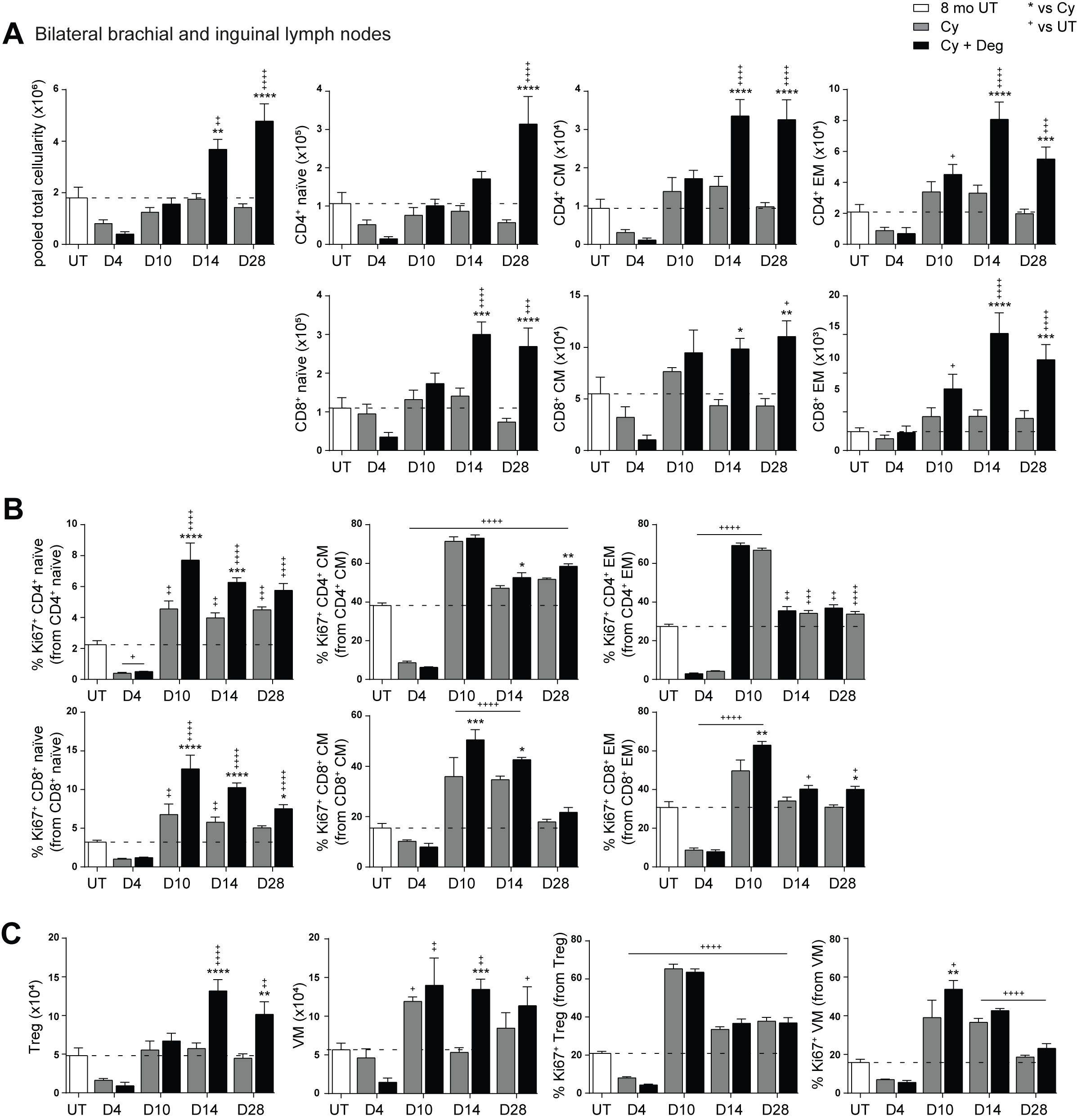


**Supplementary Figure 2.** Degarelix treatment in middle-aged male mice following cyclophosphamide damage. **(A)** Phenotypic T cell profile from pooled bilateral brachial and inguinal lymph nodes of middle-aged male mice. Total cellularity, and naïve, central memory (CM), and effector memory (EM) T cell subpopulation numbers are shown. **(B)** Proportion of Ki67^+^ T cell subsets. **(C)** Number of Tregs and virtual memory (VM) T cells; Proportion of Ki67^+^ Tregs and VM T cells. Data presented as mean ± SEM (n>3). * vs Cy, ^+^ vs UT. *p<0.05, **p<0.01, ***p<0.001, ****p<0.0001, ordinary two-way ANOVA with Sidak’s multiple comparisons.
